# HIV-1 Tat activates indoleamine 2,3 dioxygenase in murine organotypic hippocampal slice cultures in a p38 mitogen-activated protein kinase-dependent manner

**DOI:** 10.1186/1742-2094-8-88

**Published:** 2011-08-02

**Authors:** Xin Fu, Marcus A Lawson, Keith W Kelley, Robert Dantzer

**Affiliations:** 1 Integrative Immunology and Behavior Program, Department of Animal Sciences, College of ACES, University of Illinois at Urbana-Champaign, Urbana, Illinois 61801, USA; 2 Department of Pathology, College of Medicine, University of Illinois at Urbana-Champaign, Urbana, Illinois 61801-3873, USA; 3 Neuroscience Program, University of Illinois at Urbana-Champaign, Urbana, Illinois 61801, USA

## Abstract

**Background:**

We have established that activation of the tryptophan degrading enzyme indoleamine 2,3 dioxygenase (IDO) mediates the switch from cytokine-induced sickness behavior to depressive-like behavior. Because human immunodeficiency virus type 1 (HIV-1) Tat protein causes depressive-like behavior in mice, we investigated its ability to activate IDO in organotypic hippocampal slice cultures (OHSCs) derived from neonatal C57BL/6 mice.

**Methods:**

Depressive-like behavior in C57BL/6J mice was assessed by the forced swim test. Expression of cytokines and IDO mRNA in OHSCs was measured by real-time RT-PCR and cytokine protein was measured by enzyme-linked immunosorbent assays (ELISAs). p38 MAPK phosphorylation was analyzed by western blot.

**Results:**

Intracerebroventricular (*i.c.v*.) administration of Tat (40 ng) induced depressive-like behavior in the absence of sickness. Addition of Tat (40 ng/slice) to the medium of OHSCs induced IDO steady-state mRNA that peaked at 6 h. This effect was potentiated by pretreatment with IFNγ. Tat also induced the synthesis and release of TNFα and IL-6 protein in the supernatant of the slices and increased expression of the inducible isoform of nitric oxide synthase (iNOS) and the serotonin transporter (SERT). Tat had no effect on endogenous synthesis of IFNγ. To explore the mechanisms of Tat-induced IDO expression, slices were pretreated with the p38 mitogen-activated protein kinase (MAPK) inhibitor SB 202190 for 30 min before Tat treatment. SB 202190 significantly decreased IDO expression induced by Tat, and this effect was accompanied by a reduction of Tat-induced expression of TNFα, IL-6, iNOS and SERT.

**Conclusion:**

These data establish that Tat induces IDO expression via an IFNγ-independent mechanism that depends upon activation of p38 MAPK. Targeting IDO itself or the p38 MAPK signaling pathway could provide a novel therapy for comorbid depressive disorders in HIV-1-infected patients.

## Background

The risk of major depressive disorder (MDD) in human immunodeficiency virus (HIV)-infected patients is significantly greater than that in the general population [1,2]. Although highly active antiretroviral therapy (HAART) appears to suppress viral replication, there is a high level of microglial activation in the central nervous system (CNS) of the post HAART-treated patients [3], indicating that eliminating virus may not stop the process of HIV-induced ongoing inflammation in the brain. It has long been known that virus infected monocytes are able to invade the brain and induce a local inflammatory chain reaction that involves the synthesis and release of proinflammatory cytokines by infected and non-infected macrophages and microglia. In addition, infected cells can shed viral proteins such as gp120 and Tat which can activate glial cells by themselves and therefore contribute to the propagation of inflammation [4,5]. Although the production of inflammatory mediators by glial cells exposed to HIV-1 proteins has been mainly studied in the context of AIDS-related dementia [6,7], there is still limited evidence that HIV-1 proteins administered into the brain can induce depressive-like behavior in preclinical models with laboratory rodents. Rats injected with gp120 into the lateral ventricle of the brain present with signs of sickness associated with increased production of proinflammatory cytokines. Some of these behavioral alterations, such as the decreased preference for a saccharin solution, mimic the anhedonia observed in depressed patients [8,9]. Intracerebral administration of HIV-1 Tat to mice induces depressive-like behavior in mice as measured by increased immobility in the forced swim and tail suspension tests, together with increased expression of brain proinflammatory cytokines [10].

We have already established that development of inflammation-associated depression is dependent on activation of the tryptophan degrading enzyme indoleamine 2,3 dioxygenase (IDO) by proinflammatory cytokines such as IFNγ and TNFα [11,12]. IDO degrades tryptophan (Trp) into kynurenine (KYN) which can be further metabolized into neurotoxic metabolites such as 3-hydroxykynurenine and quinolinic acid (QUIN) [13]. In addition to developing depressive-like behaviors, Tat-treated mice also display increased IDO expression in their brains [10]. The occurrence of neuropsychiatric disorders in HIV-1 seropositive patients presenting with symptoms of neuro-AIDS is associated with activation of IDO in peripheral macrophages, as evidenced by decreased levels of circulating Trp and increased plasma levels of both KYN and the macrophage activation marker neopterin [14]. Confirmation that IDO activation also takes place in the brain of HIV-infected patients has been obtained since IDO enzymatic activity is increased in the brain of HIV-infected patients with HIV-associated dementia (HAD) [15]. IDO expression is up-regulated in monocytic cells in brain tissue of both patients with HIV-1 encephalitis (HIVE) [16] and monkeys infected with simian immunodeficiency virus encephalitis (SIVE) [17]. Furthermore increases in brain QUIN are associated with progression of HIV infection [18]. Brain expression of IDO during HIV infection certainly contributes to immunotolerance since administration of the IDO competitive inhibitor 1-methyl tryptophan enhances elimination of virus-infected macrophages in mice with HIVE [16].

There is evidence that HIV-1 Tat can induce IDO expression in various cell types. This is particularly the case in macrophages [19,20] but also in astrocytes exposed to HIV-1 clade B Tat [21]. We have demonstrated that organotypic hippocampal slice cultures (OHSCs) offer a reliable model for investigating neuroimmune interactions and for studying the mechanisms of IDO activation [22]. Here we have used this model to assess whether Tat alone or in conjunction with IFNγ can induce IDO expression. We chose IFNγ since this cytokine is traditionally regarded as the primary inducer of IDO [23]. IFNγ is elevated in the brains of patients with HIVE and has been hypothesized to play a role in the pathophysiology of HAD [24]. IFNγ synergizes with Tat to enhance chemokine expression, which in turn can amplify the inflammatory responses within the CNS of patients with AIDS-related neurological disorders [25]. However, IFNγ-independent activation pathway has also been reported in response to LPS in primary microglia and murine slices [22,26]. Additionally, the IFNγ-independent up-regulation of IDO expression was recently demonstrated in HIV-infected human macrophages [27]. It is unknown whether this property of IDO induction extends to OHSCs exposed to HIV proteins such as Tat. Furthermore, the precise mechanisms responsible for IFNγ-independent induction of IDO following exposure to Tat are not clearly understood.

Activation of IDO is not the sole mechanism that could be responsible for inflammation-associated depression. By reuptake of serotonin (5-HT) with high affinity in presynaptic neurons, the sodium-dependent serotonin transporter (SERT) contributes to the regulation of 5-HT neurotransmission [28]. Several cytokines, including IFNα, IFNγ, IL-1β and TNFα, are able to increase expression of SERT [29,30]. Additionally, a recent study reported that HIV-1 clade B and C Tat differentially induce SERT expression in dendritic cells [31]. For this reason, we included SERT in the possible targets of HIV-1 Tat in the *in vitro *model of OHSCs.

HIV-1 Tat mediates its biological functions by activating a variety of signaling pathways and transcription factors. The p38 mitogen-activated protein kinase (MAPK) is activated by Tat in a variety of cell types, including monocytes [32,33], macrophages [34], astrocytes [35] and the THP-1 cell line [36]. Since p38 MAPK is required for IDO expression in THP-1 cells [37], we investigated whether this signaling pathway also mediates Tat-induced expression of IDO in OHSCs. In the present study, we show that Tat induces the expression of IDO and synergizes with exogenous IFNγ to increase IDO induction in OHSCs and the effect of Tat is mediated by p38 MAPK activation.

## Methods

### Reagents

Recombinant Tat 1-72 was provided by Professor Avindra Nath through a contract with the University of Kentucky. Stock solutions of Tat 1-72 were prepared in phosphate buffered saline (PBS) (1ug/ul) and stored at -80°C until use. Recombinant murine IFNγ (cat# 315-05) was from PeproTech, Inc. Heat-inactivated horse serum (cat# SH30074.03), Hank's balanced salt solution (HBSS, cat# SH30030.03) and minimal essential media (MEM) (cat# SH30024.02) were all from Hyclone. Gey's balanced salt solution (GBSS, cat# G9779) was from Sigma, D-glucose (cat# 15023-021) was from GibcoBRL and the kits for enzyme-linked immunosorbent assays (ELISA) were obtained from R&D Systems (Wiesbaden, DE). TRIzol reagent was purchased from Invitrogen Life Technologies (Carlsbad, CA). Reagents for RT-PCR were all from Applied Biosystems as follows: high capacity cDNA reverse transcription kit (cat# 4374967); RT-PCR primers for IDO (cat# Mm00492586_m1), TNFα (cat# Mm00443258_m1), IL-6 (cat# Mm00446190_m1), the inducible isoform of nitric oxide synthase (iNOS) (cat# Mm00440485_m1) and glyceraldehyde-3-phosphate dehydrogenase (GAPDH; cat# Mm999999_g1). Antibodies specific for p38 (cat# 9212) and phosphorylated p38 (p- p38, cat# 9211) were purchased from Cell Signaling Biotechnology (Danvers, MA), whereas the secondary horseradish peroxidase (HRP)-linked rabbit anti-mouse antibody (NA934V) was obtained from GE Healthcare Biosciences (Piscataway, NJ). The p38 MAPK inhibitor SB 202190 (cat# 559397) was from EMD Chemicals, Inc. (USA). Protein was measured with a standard Bradford assay kit (cat# 500-0113, 0114, 0115) and Immun-Blot polyvinylidene difluoride (PVDF, cat# 162-0177) membranes were from Bio-RAD (Hercules, CA). ECL Western blotting detection reagents (cat# RPN2106V1, RPN2106V2) were obtained from GE Healthcare Little Chalfont (Bucks, UK).

### Mice

All animal care and use procedures were conducted in accordance with the Guide for the Care and Use of Laboratory Animals (National Research Council) and approved by the Institutional Animal Care and Use Committee. Experiments conducted *in vivo *were performed on 12-week-old male C57BL/6J mice obtained from a colony raised in our laboratory. Mice were individually housed in standard shoebox cages, with wood shavings as litter, in a temperature- (23°C) and humidity- (45-55%) controlled environment with a 12/12 h modified dark-light cycle (lights on 10:00 P.M.-10:00 A.M.). Food and water were available *ad libitum*.

### Intracerebroventricular (i.c.v.) Cannulation

Mice were surgically implanted with a single guide cannula (Plastics One, Roanoke, VA) directed toward the lateral ventricle, As previously described [10]. The guide cannulas were kept clean and covered using a screw on cannula dummy for mice (Plastics One, Roanoke, VA). Coordinates for placement of the guide cannula were 1.5 mm lateral, 0.6 mm posterior, and 1.3 mm dorsal with respect to bregma. These coordinates placed the guide cannula 1 mm dorsal to the lateral ventricle. Mice were allowed to recover 2 weeks before treatment and initiation of behavioral tests. After recovery, mice were slowly injected over 1 min *i.c.v*. with phosphate buffered saline (PBS) or Tat (40 ng) in a volume of 1 μl. This dose of Tat was selected on its ability to reliably induce IDO expression in human astrocytes [38].

### Forced swim test (FST)

The forced swim test was conducted at 24 h post *i.c.v*. injection of Tat for a five-min period and the mice were video recorded for future analysis. Immobility was defined as passive floating behavior or any movement necessary for the mouse to keep its head above water, as described previously [10].

### Organotypic hippocampal slice cultures

Murine hippocampal slice cultures were prepared using the static interface culture method [22]. Briefly, 6- to 8-day-old C57BL/6J mice were decapitated. The brains and meninges were removed, followed by separation of the hippocampus from both hemispheres. Hippocampi were dissected and transverse slices (350 μm in thickness) were prepared using a McIlwain tissue chopper (Campden Instruments Ltd, UK). Slices were placed for 1 h at 4°C into GBSS supplemented with 2 mg/ml D-glucose and were then transferred onto porous (0.4 μm) transparent membrane inserts (30-mm in diameter; Millipore) with five slices on each insert. Inserts were then placed into six-well culture plates. Each well contained 1.2 ml of nutrient medium composed of 25% heat-inactivated horse serum, 25% HBSS and 50% MEM supplemented with 25 mM D-glucose. Neither antibiotics nor anti-mycotics were used. Plates were maintained in a humidified CO_2 _incubator (5% CO_2_, 95% atmospheric air) at 37°C. Medium was changed every 2-3 days. The MEM medium was changed so that it contained only 5% horse serum and 25 mM D-glucose on the day that Tat or control medium was added. At various times following addition of Tat, supernatants were collected and stored at -80°C for measurement of cytokines. Slices were washed 3 times with cold PBS and stored at -80°C for isolation of total cellular RNA and for western blotting. Slice viability was evaluated using both propidium iodide (PI) staining and the amount of lactate dehydrogenase (LDH) released into the culture medium by CytoTox^96 ^non-radioactive cytotoxicity kit.

### Reverse transcription and real-time RT-PCR

Total cellular RNA from the hippocampus and cultured slices was extracted in TRIzol reagent, as previously described [22]. Total mRNA (1-2 μg) was reverse transcribed to cDNA using the high capacity cDNA reverse transcription kit from Ambion. Samples were analyzed in duplicate. Data were analyzed using the comparative threshold cycle method, as described elsewhere (Applied Biosystems user bulletin no.2).

### Enzyme-linked immunosorbent assays (ELISAs)

TNFα and IL-6 were measured in OHSCs supernatants with validated specific ELISA assays [22]. Briefly, 100 μl of each sample were added in duplicate to ELISA plates pre-coated with an anti-TNFα or IL-6 capture antibody. Recombinant murine TNFα and IL-6 standards ranged from 0 to 1,000 pg/ml. The lower assay limit of detection was 16 pg/ml. Absorbance was measured on an OPTImax ELISA plate reader. TNFα and IL-6 concentrations are expressed as picograms per milliliter.

### Western blot analysis

Western blotting experiments were conducted as previously described [26], with minor modifications. Briefly, slices were lysed in cold lysis buffer. Equal amounts of protein (40 μg) were separated on 10% polyacrylamide gels. Proteins were then transferred from the gel to PVDF membranes using a Bio-Rad Laboratories Mini Protein 3 system. After treating PVDF membranes with blocking buffer (TBS/0.1% Tween20 (TBST) containing 2% BSA) for 1 h at room temperature, they were incubated overnight at 4 C with blocking buffer containing primary antibodies specific for phosphorylated p38 or p38, (1:1000 dilution). Membranes were washed extensively with TBST and then incubated for 1 h at room temperature with a secondary antibody coupled to horseradish peroxidase (HRP)-conjugated sheep anti-mouse IgG antibodies at a dilution of 1:2000 in blocking buffer. Finally, membranes were washed extensively with TBST and developed with an enhanced chemiluminescence ECL Western Blot Detection Reagent. Blots were covered with transparency film and then inserted into a Fujifilm LAS-4000 System Configured for multifunctional analysis (Fujifilm, Life Science, Stamford, USA). Densitometric analysis of autoradiograms was performed using publically available IMAGE-J software from the National Institutes of Health (Bethesda, MD). Densitometric summaries were expressed as ratios of phosphorylated p38 to total p38.

### Statistical analysis

Data were analyzed using a one-way (treatment) or two-way (pretreatment × treatment) ANOVA, followed by a post hoc pairwise multiple comparison using Fischer's LSD test if the interaction was significant. All data are presented as means ± SEM. Differences were considered significant if the probability reached a level of 0.05 or less.

## Results

### Tat induces depressive-like behavior in C57BL/6J mice in the absence of sickness

**Figure 1:**
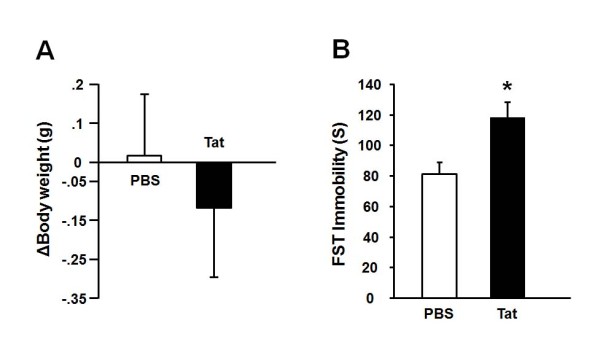
**LPS administered via the *i.c.v*. route induces depressive-like behavior**. Mice were injected *i.c.v*. with either PBS or Tat (40 ng). (A) Body weight was measured before and 24 h after injection and expressed as body weigh change. (B) Duration of immobility was quantified during a 5 min forced swim test that took place 24 h following administration of Tat or PBS. Data represent mean ± SEM (n = 6 mice/group). * *p *< 0.05 compared to PBS.

Mice were treated with *i.c.v*. PBS or Tat (40ng). At 24 h post Tat, sickness and depressive-like behavior were assessed by body weight loss and FST, respectively. Consistent with our previous findings [10], the 24 h changes in body weight did not differ according to treatment (Figure 1A, p *>*0.05). Tat-treated mice displayed increased immobility in the FST at 24 h post-treatment compared to control mice (Figure 1B, p < 0.05).

### HIV-1 Tat induces biochemical markers of inflammation-associated depression in murine OHSCs

**Figure 2:**
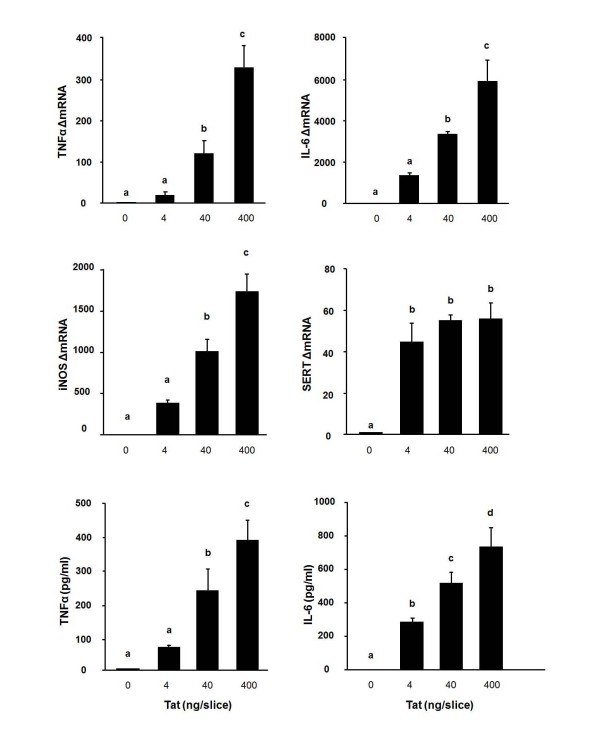
**Tat induces TNFα, IL-6, iNOS and SERT expression in OHSCs in a dose-dependent manner**. Tat at a dose of 4, 40 and 400 ng/slice was added to the medium after 10 days in culture. Tissue and media were collected 6 h later. Average Ct values for 4, 40 and 400 ng/slice Tat were, respectively, for TNFα: 22.54 ± 0.68, 19.85 ± 0.43, 18.53 ± 0.43; IL-6: 21.34 ± 0.21, 19.88 ± 0.03, 19.29 ± 0.12; iNOS: 21.69 ± 0.14, 19.92 ± 0.14, 19.42 ± 0.26; SERT: 28.45 ± 0.04, 27.91 ± 0.02, 28.09 ± 0.15. Levels of TNFα and IL-6 (pg/ml) were measured in the supernatant by ELISA. Data represent the mean ± SEM (n = 3 in each group). Bars labeled with different letters (a, b, c or d) are significantly different from each other at *p *< 0.05.

We recently found that central injection of HIV-1 Tat (40 ng) increased brain IDO and cytokine steady-state transcripts at 4 h [10]. Here we determined whether the expression of IDO and cytokines response to Tat occurs in murine OHSCs as it does in the CNS *in vivo*. Based on the *in vivo *response to central injection of Tat [10], the time point of 6 h was selected for carrying out dose-response experiments to determine the effect of Tat on cytokine and IDO expression. Slices were exposed to 4, 40 and 400 ng/slice of Tat on day 10 in culture. As shown in Figure 2, Tat significantly increased TNFα, IL-6 and iNOS at the mRNA level (*p *< 0.05) in a dose-dependent manner, with a maximum at 400 ng/slice. Tat-induced increases in TNFα and IL-6 mRNA expression were paralleled by a concomitant increase in protein production (Figure 2, *p *< 0.05). Increased iNOS mRNA was not associated with any detectable increase in nitrite levels at the 6 h time point (data not shown). Induction of SERT mRNA reached a maximum at 4 ng/slice (Figure 2, *p *< 0.05).

**Figure 3:**
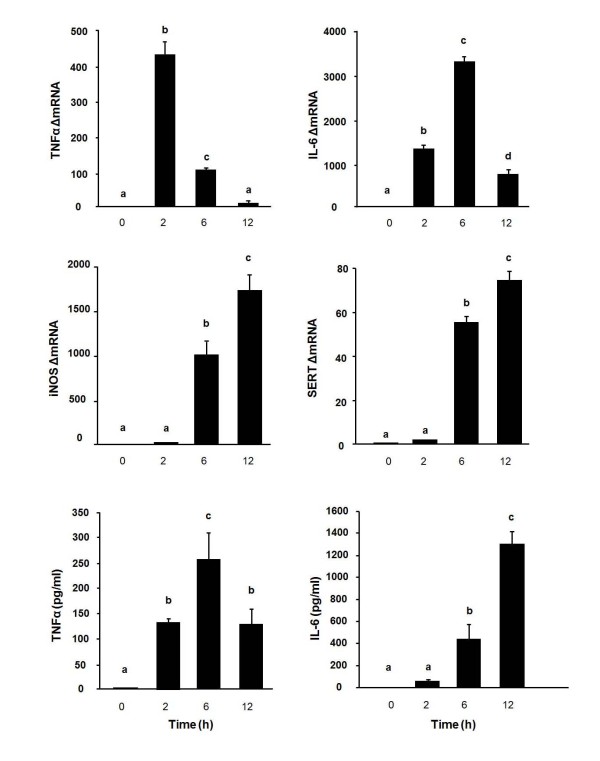
**Tat induces TNFα, IL-6, iNOS and SERT expression in OHSCs in a time-dependent manner**. Hippocampal slices were treated with Tat (40 ng/slice) and tissue and media were collected at various times. Average Ct values at 2, 6 and 12 h were, respectively, for TNFα: 18.24 ± 0.76, 20.42 ± 0.75, 23.32 ± 1.01; IL-6: 21.08 ± 0.19, 19.88 ± 0.03, 21.98 ± 0.31; iNOS: 28.47 ± 1.91, 19.92 ± 0.14, 19.08 ± 0.25; SERT: 32.42 ± 0.28, 27.91 ± 0.02, 27.50 ± 0.18. Levels of TNFα and IL-6 (pg/ml) were measured in the supernatant by ELISA. Data represent the mean ± SEM (n = 3 in each group). Bars labeled with different letters (a, b, c or d) are significantly different from each other at *p *< 0.05.

**Figure 4:**
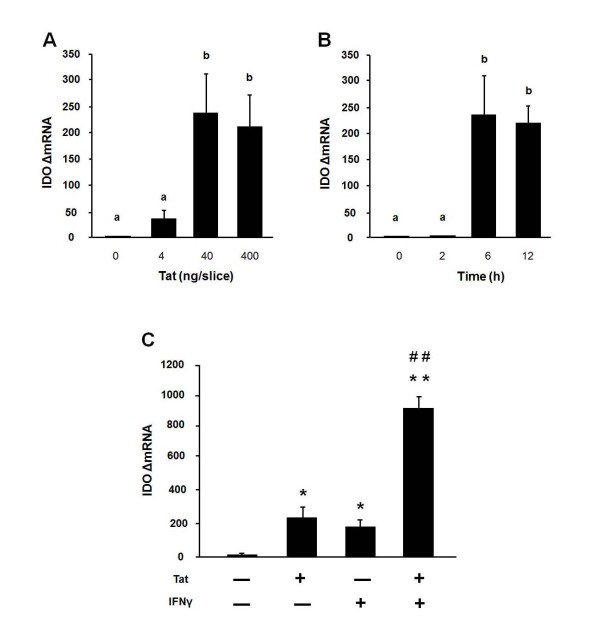
**Tat induces IDO expression in OHSCs and this effect is potentiated by pretreatment with IFNγ**. (A) 4, 40 and 400 ng/slice Tat were added to the medium after 10 days in culture. Tissue was collected 6 h following addition of Tat. Average Ct values for 4, 40 and 400 ng/slice Tat for IDO were, respectively, 35.54 ± 0.29, 32.60 ± 0.39, 32.92 ± 0.30. (B) Hippocampal slices were treated with Tat (40 ng/slice) for 2, 6 and 12 h. Average Ct values at 2, 6 and 12 h for IDO were, respectively, 40.00 ± 0.00, 32.60 ± 0.39, 32.61 ± 0.25. Bars represent the mean ± SEM (n = 3 in each group). Bars labeled with different letters (a or b) are significantly different from each other at *p *< 0.05. (C) Slices were preincubated with medium alone or with IFNγ (10 ng/ml) for 24 h, and this was followed by addition of 40 ng/slice Tat. Slices were collected 6 h later. Average Ct values for Tat, IFNγ and Tat + IFNγ were, respectively: 32.54 ± 0.37; 32.56 ± 0.25; 29.76 ± 0.37. Data represent the mean ± SEM (n = 3 in each group). Bars labeled with different letters (a or b) are significantly different from each other at *p *< 0.05. * *p *< 0.05, ** *p *< 0.01 compared to medium control; ## *p *< 0.01 compared to Tat or IFNγ treatment.

For kinetic studies, OHSCs were exposed to 40 ng/slice Tat for 2, 6 and 12 h on day 10 of culture. As shown in Figure 3, the greatest expression of both mRNA and protein for TNFα occurred at 2 h (*p *< 0.05) and 6 h (*p *< 0.05), respectively. IL-6 mRNA peaked at 6 h (*p *< 0.05) and gradually decreased at 12 h. IL-6 concentration in the culture medium increased after 6 h (*p *< 0.05) and reached a maximum at 12 h (*p *< 0.05). iNOS and SERT mRNA increased at 6 h (*p *< 0.05) and peaked at 12 h (*p *< 0.05), but there was no concomitant increase in nitrite levels at any time point (data not shown). We also used real-time RT-PCR to determine whether Tat induces IDO steady-state transcripts in OHSCs as it does *in vivo*. To determine an optimal dose for Tat-induced expression of IDO, OHSCs were treated with 4, 40 and 400 ng/slice of Tat for 6 h. As shown in Figure 4A, IDO mRNA expression could be detected at 4 ng/slice Tat and peaked at 40 ng/slice Tat (*p *< 0.05). Kinetic studies were then carried out in which OHSCs were exposed to Tat (40 ng/slice) for 2, 6 and 12 h. As shown in Figure 4B, IDO mRNA could not be detected in OHSCs prior to addition of Tat (40 amplification cycles). However, IDO expression was significantly induced by Tat at 6 h (*p *< 0.05) with no further increase at 12 h. This effect was similar in intensity to that of exogenous IFNγ (10 ng/ml, Figure 4C, p < 0.05). Moreover, pretreatment of OHSCs with the same dose of IFNγ 24 h before Tat markedly amplified IDO responses to Tat (Figure 4C, p < 0.01). However, the Tat-induced expression of IDO did not require endogenous synthesis of IFNγ because no IFNγ mRNA could be detected at 6 h in Tat-stimulated slices (40 amplification cycles, data not shown).

### p38 MAPK is required for HIV-1 Tat-induced IDO expression

**Figure 5:**
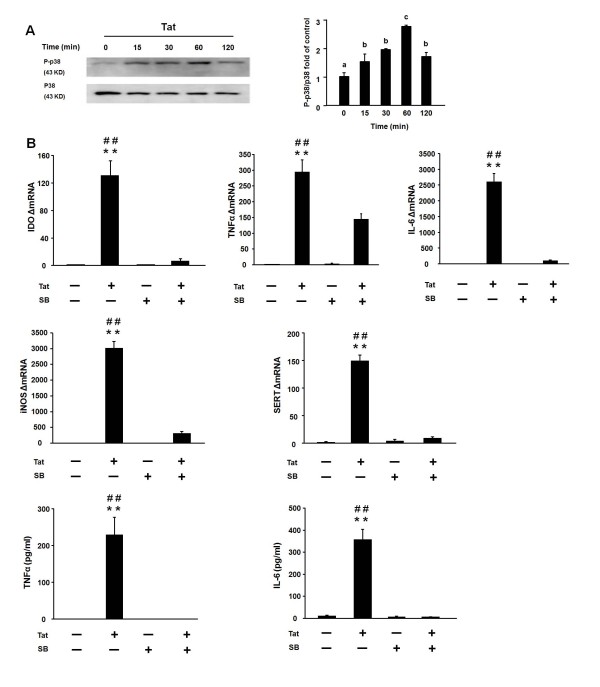
**p38 MAPK activation mediates Tat-induced expression of proinflammatory cytokines, iNOS, SERT and IDO in OHSCs**. (A) Tat induces Thr180/Tyr182 phosphorylation of p38 MAPK MAPK as early as 15 min with a maximum at 60 min in OHSCs. Hippocampal slices were treated with Tat (40 ng/slice) at 15, 30, 60 and 120 min, and cell lysates were collected for p38 MAPK phosphorylation analysis by Western blot. A representative Western blot showing results with hippocampal slices incubated with Tat at the above time points is shown, followed by densitometric analysis of Western blots from three independent experiments. Densitometric data were calculated as ratio of phosphorylated p38 MAPK to total p38 MAPK. Bars labeled with different letters (a, b or c) are significantly different from each other at *p *< 0.05. (B) The p38 MAPK inhibitor, SB 202190, abrogates Tat-induced expression of IDO, iNOS, SERT and proinflammatory cytokines. Hippocampal slices were treated with SB 202190 (30 μM) for 30 min and then incubated with or without Tat (40 ng/slice) for 6 h. SB 202190 inhibited Tat-induced IDO, iNOS, SERT, TNFα and IL-6 expression. Data represent the mean ± SEM (n = 3 in each group). ** *p *< 0.01 compared to medium control; *## p *< 0.01 compared to Tat + SB treatment. SB = SB 202190.

In accordance with the results of others on the effects of Tat on p38 MAPK in various cell types [32-36], we confirmed that HIV-1 Tat can activate p38 MAPK in OHSCs. Slices were treated with Tat for 15, 30, 60 and 120 min respectively and the lysates were analyzed for phospho-p38 activity by Western blot analysis. We found that Tat induced significant phosphorylation of p38 MAPK as early as 15 min (Figure 5A, p < 0.05) with a maximum at 60 min (Figure 5A, p < 0.05).

To determine whether p38 MAPK is involved in Tat-induced IDO expression, the p38 MAPK inhibitor SB 202190 was employed. We previously established that 30 μM SB 202190 significantly suppresses cytokine expression at both the mRNA and protein levels in response to LPS stimulation (data not shown). The dose of 30 uM was therefore selected for further experiments on Tat-induced IDO expression. Slices were pretreated with SB 202190 (30 μM) for 30 min before stimulation with Tat for another 6 h. SB 202190 abrogated the Tat-induced expression of IDO, IL-6, iNOS and SERT transcripts, although this effect was only partial for TNFα mRNA (Figure 5B, p < 0.01). SB 202190 also fully blocked the Tat-induced release of cytokine proteins in the slice supernatants (Figure 5B, p < 0.01). Importantly, neither Tat nor SB 202190 affected viability of the cells, as determined by measuring both PI staining and release of lactate dehydrogenase into the culture medium (data not shown). These data clearly demonstrate that the p38 MAPK signaling pathway is necessary for the Tat-induced expression of both proinflammatory cytokines and IDO.

## Discussion

Results of the present experiments establish that HIV-1 Tat induces depressive-like behavior *in vivo *and induces production of proinflammatory cytokine *in vitro *in murine OHSCs and increases expression of IDO and SERT. These changes are dependent on activation of the p38 MAPK signaling pathway.

Our *in vivo *data show that Tat increases the duration of immobility in the forced swim test in mice without inducing any sickness, as measured by the lack of body weight loss. These data are in agreement with already published findings from our group on depressogenic activity of Tat [10]. The mechanisms of the depressogenic activity of Tat were further investigated *in vitro*. Compared to primary cultures of brain cells, OHSCs have the advantage of preserving the cellular and connective organization as well as several fundamental *in vivo*-like characteristics such as glial-neuronal interactions [39,40]. Although this preparation has previously been used for studying the detrimental effects of proinflammatory cytokines on long-term potentiation in the rat system [41,42], OHSCs have rarely been used for the investigation of neuroimmune interactions. Our previous experiments demonstrated that long term culture of OHSCs can be reliably used to study neuroimmune mechanisms of induction of IDO [22]. We therefore employed OHSCs in the present experiments to study the mechanisms involved in the expression of IDO by Tat.

Depression is an important comorbid condition of HIV infection. We previously demonstrated that IDO, the first and rate-limiting enzyme in the synthesis of KYN from the precursor of Trp, is both sufficient and necessary to mediate depressive-like behavior in response to either acute or chronic activation of the immune system in mice [43,44]. Moreover, the increase in brain IDO activity is invariably preceded by enhanced expression of IDO mRNA, which can therefore be used as a surrogate marker of IDO activation [26,43,44]. Therefore, in the present study, we examined the effects of Tat on IDO mRNA in OHSCs. As shown in Figure 2, HIV-1 Tat protein significantly up-regulates IDO mRNA expression in OHSCs. These results are consistent with previous studies that describe Tat-mediated induction of the IDO expression in other types of cells [19-21]. These *in vitro *data are also in accordance with our *in vivo *results showing that Tat induced depressive-like behavior is associated with increased expression of IDO in the brain [10]. Taken together, these data provide the first evidence to indicate that activation of IDO in response to Tat stimulation in the brain could be a key event in the switch from sickness to depressive-like behavior.

IFNγ is considered to be the prototypical inducer of IDO in a variety of cells [23] as well as in clinical situations in which inflammation-associated depression occurs [45]. Additionally, IFNγ is increased in the brain during HIV infection [24] and synergizes with Tat to play a critical role in the pathogenesis of HAD [25]. It is well documented that monocytes/macrophages/microglia can produce IFNγ [46-48]. However, IDO expression appears to be up-regulated in an IFNγ-independent manner in HIV-infected human macrophages [27]. IFNγ protein remained undetectable in HIV-infected human macrophage supernatants while IDO expression increased [27]. Our data indicate that IDO induction by Tat does not necessarily require synthesis of IFNγ because no IFNγ transcripts could be detected at 6 h. These observations are in accordance with the results obtained by Boasso et al. [49]. Boasso et al. reported that blockade of either type I or type II IFNs by antibodies was ineffective in preventing the induction of IDO in human peripheral blood mononuclear cells exposed to R5- or X4-trophic HIV. Therefore, our data extend the developing concept that IDO can be induced by Tat in an IFNγ-independent mechanism.

Addition of Tat to OHSCs induced the synthesis and release of proinflammatory cytokines. These cytokines are well known to stimulate HIV-1 replication and contribute to HIV pathogenesis [50,51]. Increased expression of proinflammatory cytokines in the brain is observed in HIV-1 infected patients [52]. Tat also can up-regulate cytokine expression, such as IL-1β and TNFα, in peripheral blood macrophages, CNS-derived cell lines and primary astrocytes, microglia [53,54] and human monocytes [55]. The large increase in proinflammatory cytokine expression could mediate IDO induction by Tat. Both *in vivo *and *in vitro *studies have shown that IDO induction is associated to IL-1β. For instance, pretreatment *in vivo *with the anti-inflammatory tetracycline derivative minocycline attenuates LPS-induced expression of brain IL-1β, indicating that IL-1β probably participates in LPS-induced expression of brain IDO [44]. A synergistic activation of IDO by IL-1β, TNF-α and IL-6 has been reported in human monocytic THP-1 cells exposed to LPS [37]. We observed that IL-1β mRNA can be induced by Tat in OHSCs (data not shown). However, this induction does not lead to release of IL-1β protein if exogenous ATP is not added to the culture. This is because ATP is necessary for processing and release of the mature IL-1β protein [56]. In the absence of IL-1β, the most likely mediators of IFNγ-independent IDO induction in OHSCs are TNF-α and IL-6. IDO induction has been shown to be mediated mainly by TNF-α, but not by IL-6 in human monocytic THP-1 cell cultures exposed to immune stimulation [57]. However, IL-6 can synergize with TNF-α to increase IDO activity [37]. The exact cytokine signaling pathways that are predominantly involved in the production of IDO in response to Tat in OHSCs remain to be elucidated.

In addition to IDO induction by Tat, we observed a robust induction of SERT mRNA in response to Tat. This finding indicates that OHSCs can serve as a reliable *in vitro *model for investigating the possible contribution of serotonin re-uptake mechanisms in comorbid depressive disorders in HIV-infected patients. Whether this change at the mRNA level translates into functional changes in SERT remains to be established. This is an important perspective since differences in the expression and function of SERT are well known to affect many human and mouse quantitative traits, including anxiety- and depression-related behaviors [58].

To elucidate the signaling pathways that mediate Tat-induced IDO expression, we examined whether Tat contributes to overexpression of IDO by activating p38 MAPK. This signaling pathway has been shown to be required for IDO expression in THP-1 cells following LPS stimulation [37]. Moreover, HIV-1 Tat protein has been reported to activate p38 MAPK in a variety of cells, including monoctyes [32,33], macrophages [34], astrocytes [35] and the human THP-1 cell line [36]. We therefore tested the possibility that Tat-induced p38 promotes expression of IDO in OHSCs. SB 202190, a highly selective, potent and cell permeable inhibitor of p38 MAPK [59], was employed to inhibit p38 activation. It binds within the ATP pocket of the active kinase with a K_d _of 38 nM, as measured in recombinant human p38, and selectively inhibits both the p38α and β isoforms. We observed that SB 202190 significantly inhibited Tat-induction of IDO in OHSCs, which is consistent with the possibility that p38 may be involved in the development of Tat-induced depressive-like behavior.

The demonstration of an inhibitory effect of SB 202190 on Tat-induced changes in OHSCs does not mean p38 MAPK signaling is directly responsible for these effects. Activation of p38 MAPK could act indirectly via NF_k_B activation [60,61] or AP-1 activity at both the transcriptional and post-transcriptional levels [62]. The human IDO promoter region contains multiple AP-1 and NF_k_B sites [37]. Therefore, the possibility that other transcription factors are involved in Tat-induced IDO expression cannot be dismissed. However, our data clearly demonstrate that Tat-induced IDO expression in OHSCs is mediated, at least in part, through a p38-dependent mechanism.

In conclusion, results of the present studies demonstrate that p38 MAPK is potently involved in HIV-1 Tat-induced IDO expression. These studies provide further evidence for targeting the brain IDO and p38 MAPK signaling pathway in the treatment of depressive disorders associated with HIV infection.

## Competing interests

RD has received honorarium from Astra-Zeneca, Bristol-Myers-Squibb, Janssen and Lundbeck Laboratories. He is working as a consultant for Lundbeck Laboratories. KWK has received honorarium from Astra-Zeneca.

## Authors' contributions

XF designed the experiments with the help of KWK and RD, performed the *in vitro *component of theseexperiments, analyzed the results and drafted the manuscript. MAL was responsible for performing and analyzing the *in vivo *component of these experiments. RD and KWK secured funding for the project and helped with the final version of the manuscript. All authors read and approved the final manuscript.
